# Glycemic and lipid responses to selenium-enriched vs. zeaxanthin-enriched eggs in patients with type 2 diabetes: a 12-week randomized controlled trial

**DOI:** 10.3389/fnut.2026.1803814

**Published:** 2026-07-10

**Authors:** Xiaokang Niu, Yong Zhang, Yinghua Liu, Yinhua Zhu, Bing Fang, Chenghao Hu, Xiaojuan Xu, Ran Wang, Yue Sang, Qi Zhang, Jie Guo

**Affiliations:** 1 Research Center for Probiotics, Department of Nutrition and Health, China Agricultural University, Beijing, China; 2 Department of Nutrition, The First Medical Center of Chinese PLA General Hospital, Beijing, China; 3 Henan Layer Breeding Engineering Technology Research Center, Jiyuan, China

**Keywords:** egg, randomized controlled trial, selenium, type 2 diabetes mellitus, zeaxanthin

## Abstract

**Background/objectives:**

The present study investigated the effect of selenium-enriched eggs (SE egg) vs. zeaxanthin-enriched eggs (ZE egg) on glycemic and lipid metabolism in patients with diabetes.

**Methods:**

Fifty-eight patients with type 2 diabetes were recruited and randomly divided into group SE egg (27 subjects) and ZE egg (31 subjects). The two groups of participants consumed either SE egg or ZE egg twice a day for a total of 12 weeks of intervention. Fasting blood samples were collected before and after the intervention to detect changes in blood glucose and lipid levels.

**Results:**

After the intervention, the fasting blood glucose, homeostatic model assessment of insulin resistance, and triglyceride-glucose index of both SE and ZE groups of subjects significantly decreased (*p* < 0.05). Among subjects with a baseline glycosylated hemoglobin A1c (HbA1c) < 8.9%, eggs from both groups showed an enhanced improvement in glucose and lipid metabolism. The ZE egg group showed a significant decrease in triglycerides (*p* = 0.0007) and an increase in homeostatic model assessment of *β*-cell function (*p* = 0.06). There was no significant difference in HbA1c between the two groups after the intervention.

**Conclusion:**

This study demonstrates that SE egg and ZE eggs have comparable effects on improving glucose metabolism profiles in diabetic patients, with an improved efficacy observed in subjects with lower baseline HbA1c levels. Furthermore, ZE eggs suggest potential benefits in lowering TG levels.

**Clinical trial registration:**

https://www.chictr.org.cn/index.html, Identifer, ChiCTR2300077522.

## 1 Introduction

As a globally prevalent chronic metabolic disorder, type 2 diabetes mellitus (T2DM) manifests as sustained hyperglycemia, largely attributed to a combination of insulin resistance and *β*-cell dysfunction (1). This dysregulation of glucose metabolism is commonly accompanied by lipid abnormalities, such as elevated triglycerides (TG), low-density lipoprotein cholesterol (LDL-C), total cholesterol (TC), and reduced high-density lipoprotein cholesterol (HDL-C), which together constitute a major cardiovascular risk factor (2). Consequently, identifying comprehensive interventions capable of simultaneously ameliorating glucose and lipid metabolism has become a central focus of nutritional research in the field of diabetes.

Current therapeutic strategies for T2DM primarily include pharmacological treatments (e.g., sulfonylureas and glinides) and lifestyle modifications (e.g., a healthy diet and physical activity) (3–5). However, accumulating evidence indicates that pharmacotherapy is often accompanied by adverse events, such as hypoglycemia, dementia, and gastrointestinal intolerance (6, 7), whereas lifestyle interventions, such as structured physical activity, are often limited by poor long-term adherence and modest efficacy (8, 9). This underscores the necessity of developing safer, sustainable approaches for achieving long-term metabolic health in diabetes care.

Dietary modification remains a cornerstone of diabetes management (10, 11). Eggs are rich in several key nutrients, including bioavailable protein, monounsaturated fatty acids, phospholipids, and a range of essential micronutrients, and they represent a relevant component of this dietary framework (12). However, their high cholesterol content, particularly in the yolk, has prompted a longstanding debate regarding their metabolic effects, especially on lipid profiles in individuals with diabetes (13, 14). The nutrient composition of eggs is not fixed but is markedly shaped by the diet of laying hens. Functional eggs produced through hen feed fortification may provide specific metabolic benefits beyond those of ordinary eggs (15–17). Many components in eggs, such as lutein and vitamin E, have been demonstrated to improve glucose and lipid metabolism (18). Selenium primarily via the selenoprotein glutathione peroxidase 1, improves insulin sensitivity by modulating the redox balance in insulin-sensitive tissues such as the liver and muscle (19). Selenoproteins can enhance insulin signaling by inactivating protein tyrosine phosphatases through the regulation of reactive oxygen species, thereby promoting glucose uptake (20). Selenium has been shown to reduce fasting blood glucose and improve insulin resistance in diabetic patients (21–25). Evidence suggests that zeaxanthin plays a potential role in alleviating diabetic conditions, indicating its therapeutic implications for metabolic management (26). Zeaxanthin exerts antihyperglycemic effects by activating the Nrf2-mediated antioxidant pathway (27). Furthermore, zeaxanthin reduces plasma sugar level via the induction of PPAR-*γ* expression and modulates the immune system activity through decrease of chemokines and cytokines to improve the symptoms of hyperglycemia (28). However, evidence regarding the potential of selenium- and zeaxanthin-enriched eggs to ameliorate diabetic symptoms remains limited.

To date, most studies have focused on the binary question of egg consumption vs. non-consumption in patients with diabetes, yielding inconsistent results. However, few studies have explored whether optimizing the intrinsic nutrient profile of eggs could provide targeted metabolic benefits. To address this, the present randomized controlled trial conducted a head-to-head comparison of selenium-enriched eggs and zeaxanthin-enriched eggs. This approach aims to elucidate the distinct physiological effects of these two functional enrichments on glycemic and lipidemic indices in adults with T2DM, thereby providing evidence for personalized dietary interventions.

## 2 Materials and methods

### 2.1 Egg composition detection

The selenium, zinc, lutein, zeaxanthin content in eggs was detected by the method described in the National food safety standards of the people’s Republic of China.

### 2.2 Study design

A randomized, double-blind, controlled trial was implemented to assess the effects of SE eggs and ZE eggs on glucose and lipid metabolism parameters in diabetic individuals in Beijing, China. The protocol received approval from the Institutional Review Board of the China Agricultural University Ethics Committee, confirming adherence to ethical standards. (approval code: CAUHR-20231101; approval date: 2023/11/01). All participants provided written informed consent prior to enrollment. The study was registered at Chictr.org.cn with the registration number of ChiCTR2300077522 (Date of registration: 2023/11/10).

### 2.3 Inclusion and exclusion criteria

Inclusion criteria: patients (1) age over 18 years; (2) with type 2 diabetes diagnosed in a hospital; (3) with a history of diabetes exceeding 3 years; (4) had either a glycosylated hemoglobin A1c (HbA1c) ≥ 6.5% or a fasting blood glucose (FBG) ≥ 7.0 mmol/L.

Exclusion criteria: patients (1) have any other types of diabetes; (2) suffering from genetic syndromes/disorders affecting glucose tolerance (including anemia, hemoglobinopathy, etc.); (3) have a documented history of severe psychiatric disorders; (4) presenting with severe comorbidities; (5) have undergone cardiac surgery within the past 3 months; (6) have a history of alcohol abuse or substance misuse in the last year; (7) currently on medications that may interfere with the study outcomes; (8) recent use (within 30 days) of medications affecting insulin sensitivity/secretion; (9) recent use (within 30 days) of medications known to induce weight gain; (10) recent use (within 30 days) of weight-loss medications or participation in weight-loss programs; (11) currently using medications that affect the metabolism of the investigational product; (12) lactating, pregnant, or planning pregnancy during the trial; (13) engaged in shift work or having irregular schedules that could hinder intervention adherence; (14) physically unable to comply with the lifestyle intervention; (15) having food allergies/intolerances relevant to the study; (16) participated in another clinical trial within the last 30 days or currently enrolled in multiple trials.

### 2.4 Randomization and blinding

The randomization process was performed by an independent research assistant who was not involved in the trial. A simple random sequence was generated via computer software. To ensure allocation concealment, the assignments were placed in sequentially numbered, sealed, opaque envelopes. Upon recruitment and enrollment, the independent assistant opened the envelopes in consecutive order to assign participants (at a 1:1 ratio) to either the SE egg group or the ZE egg group. A double-blind design was maintained, whereby both the researchers and participants remained unaware of the intervention assignments until study completion. To ensure that the eggs were indistinguishable, both the SE and ZE eggs were produced using standardized farming protocols to ensure identical size, shell color, and yolk pigmentation. Before distribution, the eggs were cleaned and packaged in identical, plain white cartons labeled only with unique coded identifiers (Group A and Group B).

### 2.5 Intervention and procedures

Prior to the commencement of the trial, eligible participants were recruited through online platforms and screened based on predefined inclusion criteria, including age, sex, FBG, HbA1c, body composition, disease status, and medication use. During the 12-week intervention, participants were instructed to consume their assigned type of eggs (SE egg or ZE egg) twice daily, before breakfast and dinner. Each whole egg was beaten, dropped into plain boiling water, and boiled until fully cooked. The participants then allowed the mixture to cool to room temperature and consumed it as a drink. Absolutely no cooking oils, salt, or other seasonings were added. Because participants were thoroughly briefed on this scientific necessity prior to enrollment, and the formulation was generally well-tolerated. Throughout the intervention, we actively monitored gastrointestinal tolerance and palatability via weekly follow-ups. They were advised to maintain their usual dietary and physical activity habits and to avoid consuming additional egg-based foods or supplements throughout the study. Patients were required to have maintained a stable dosage and regimen of any prescribed hypoglycemic or lipid-lowering medications for at least 3 months prior to enrollment, with no anticipated plans to change these regimens during the study period. They were also required to return empty egg cartons at each follow-up visit and record their daily intake in a dedicated WeChat group.

### 2.6 Dietary and physical activity assessment

Food frequency questionnaire was administered to all participants at randomization. Participants were asked how often, on average, they had consumed each food item during the preceding year. The average daily step count and the frequency of daily exercise were also recorded for all subjects.

### 2.7 Body weight measurement

Body weight was measured using the Inbody 770 body composition tester (Biospace Shanghai Co., Ltd., China).

### 2.8 Blood sample collection and blood parameters measurement

All blood samples were collected while the participants were fasting. FBG was detected by using a glucose meter (F. Hoffmann-La Roche, Ltd., Switzerland). HbA1c was detected by a glycated hemoglobin analyzer (Arkray Co., Ltd., Japan). Venous blood samples were collected at baseline (Week 0) and at 4-week intervals throughout the intervention (Weeks 4, 8, and 12) to monitor changes in glycemic control. FBG was measured at each of these time points. Serum samples were collected at baseline and after the intervention period, and blood lipids, insulin, and liver and kidney function indicators were quantitatively analyzed.

TC, TG, and HDL-C were measured using colorimetric test kits (Jiangsu Meimian industrial Co., Ltd., China). LDL-C was measured using a two-reagent direct method kit (Jiangsu Meimian Industrial Co., Ltd., China). Insulin was measured using ELISA kits (Beijing Solarbio Science & Technology Co., Ltd., China). To assess safety, the levels of serum uric acid (UA), creatinine (CR), urea (UR), aspartate aminotransferase (AST), and alanine aminotransferase (ALT) were analyzed using ELISA kits (Elabscience Biotechnology Co., Ltd., China). All biochemical analyses were performed in duplicate to ensure precision. The intra-assay and inter-assay coefficients of variation for all measured parameters were less than 5.0%. Regular quality control procedures were implemented using standard reference materials, and all results were within the predefined acceptable limits. The homeostasis model assessment of insulin resistance (HOMA-IR) index, Homeostasis model assessment of *β*-cell function (HOMA-β) index and Triglyceride-glucose (TyG) index were calculated using the following formulas (29, 30):


$$\text{HOMA}-\mathrm{IR}=\frac{\text{fasting insulin}\;(\mathrm{mIU}/L)\times \text{fasting glucose}\;(\text{mmol}/L)}{22.5}$$

$$\text{HOMA}-\beta =\frac{20\times \text{fasting insulin}\;(\mathrm{mIU}/L)\;}{\text{fasting glucose}\;(\text{mmol}/L)-3.5}$$

TyG=ln[triglyceride(mg/dL)×fasting glucose(mg/dL)/2]

Notes: For the calculation of the TyG index, fasting blood glucose values were converted from mmol/L to mg/dL using the conversion factor of 1 mmol/L = 18 mg/dL. Triglyceride levels were converted from mmol/L to mg/dL by multiplying by 88.5.

### 2.9 Outcomes

The primary outcome was HbA1c and FBG. The secondary outcomes included metabolic profile (insulin, HOMA-IR, HOMA-*β*, and TyG index), lipid profile (TG, TC, LDL-C, and HDL-C), and safety indicators (AST, ALT, UA, CR, and UR).

### 2.10 Statistical analysis

For participants who discontinued the intervention or were lost to follow-up, missing data were handled using the Intention-to-Treat principle with the last observation carried forward method. Continuous variables are reported as the mean ± standard deviation if normally distributed; otherwise, median and interquartile range are used. For comparisons, paired t-tests or paired-sample rank-sum test were applied within groups, while independent t-tests or Mann–Whitney tests were utilized for between-group analyses. Multiple testing was performed using the Bonferroni method. Linear mixed models were used to analyze outcomes measured at multiple time points. In the initial proposal, we did not include a pre-specified subgroup analysis. *Post-hoc* subgroup analyses were conducted based on specific clinical thresholds of HbA1c (8.9%) and TG (1.7 mmol/L) to explore potential signal variations. The sample size was estimated based on the primary outcome measure HbA1c. According to the results of the pilot study, the difference in HbA1c levels between the control and intervention groups was 0.63%, with a corresponding standard deviation of approximately 1.12%. Assuming a power of 80%, a significance level of 0.05, and an expected 15% dropout rate, 30 participants were required to be recruited for each treatment group, resulting in a total sample size of 60 participants. To clarify the statistical reliability of the secondary outcomes, group-specific *post-hoc* power analyses were performed using G*Power software (version 3.1.9.7) for intra-group changes (matched pairs, two-tailed, *α* = 0.05, assuming a conservative pre-to-post correlation of 0.50). The achieved statistical power for the HOMA-IR index was 0.77 for the SE egg group and 0.62 for the ZE egg group. For triglycerides (TG), the achieved power was 0.05 for the SE egg group and 0.28 for the ZE egg group. Analyses were performed using SPSS 25. Figures were generated using GraphPad Prism 9, with statistical significance set at a *p*-value < 0.05.

## 3 Results

### 3.1 Nutritional composition of eggs

**Figure 1:**
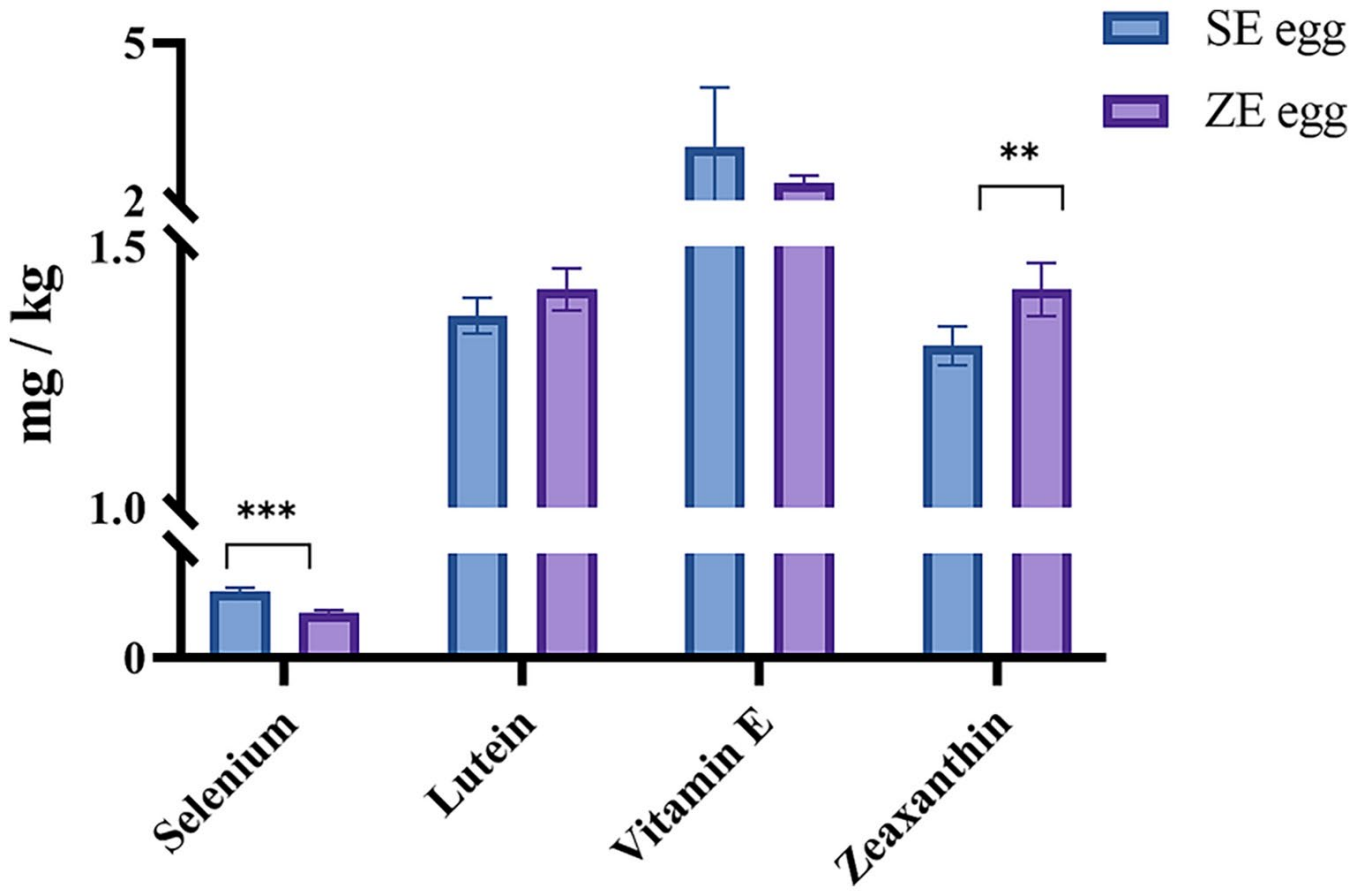
Nutritional composition of eggs. SE egg, selenium-enriched egg; ZE egg, zeaxanthin-enriched egg.

Figure 1 presents the nutritional composition of the two different types of eggs. The selenium content was significantly higher in SE eggs than in ZE eggs (317.4 μg/kg egg vs. 217.6 μg/kg egg, *p* < 0.001). In contrast, ZE eggs contained significantly higher levels of zeaxanthin (1.42 mg/kg egg vs. 1.31 mg/kg egg, *p* < 0.01). Based on these absolute concentrations and the consumption of two eggs per day, the actual daily intake was calculated as follows: the SE group consumed 31.74 μg of selenium and 131 μg of zeaxanthin per day, and the ZE group consumed 21.76 μg of selenium and 142 μg of zeaxanthin per day.

### 3.2 Baseline characteristics

**Figure 2:**
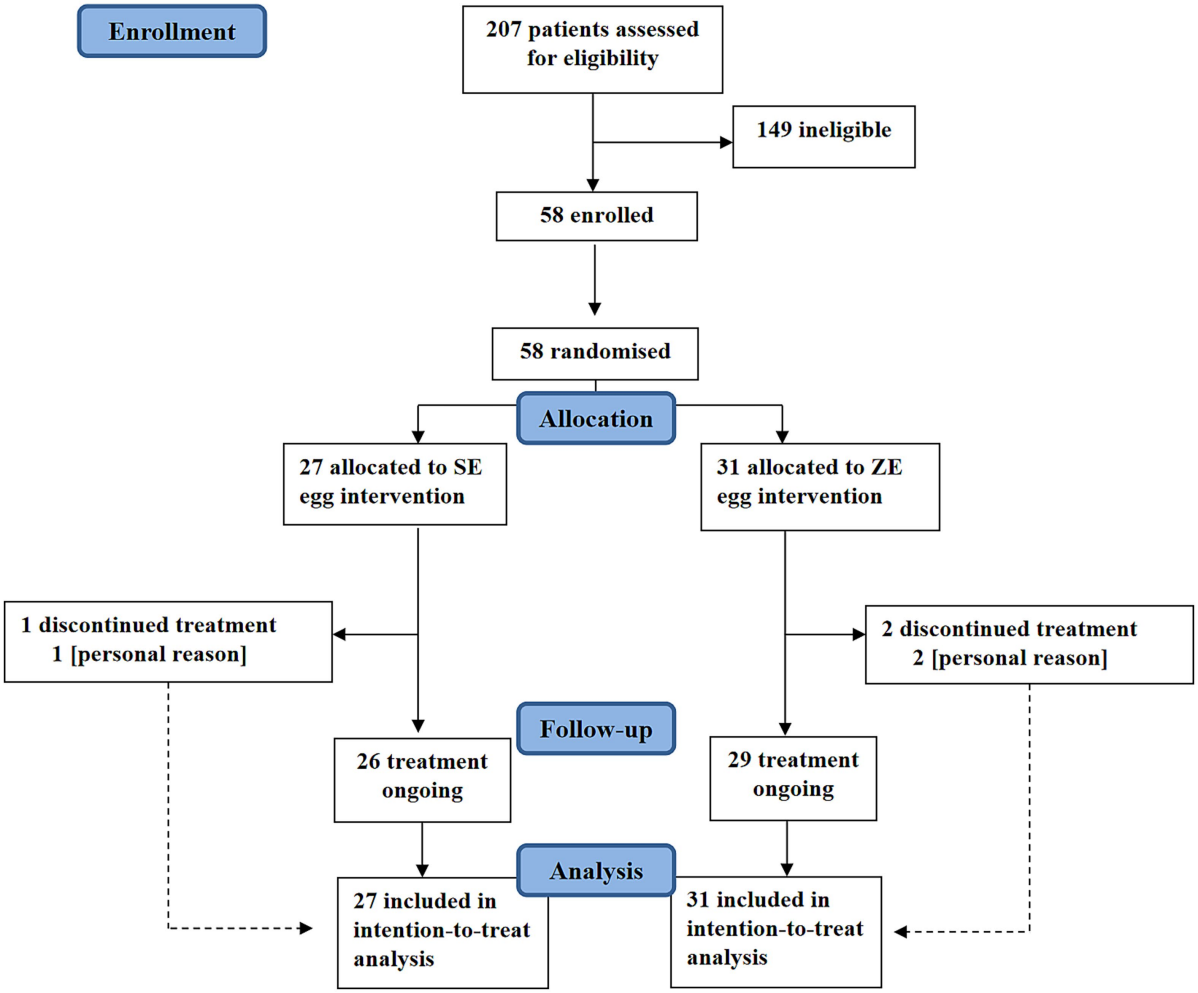
Flow of participants through the study.

The study was carried out as a randomized, double-blind, controlled trial between November 2023 and April 2024. Initially, 207 subjects were recruited for screening; finally, 58 of them satisfied the inclusion criteria and were included (Figure 2). Participants were randomized to the SE egg or ZE egg group. Three subjects discontinued due to personal reasons, with 55 completing the trial representing an overall adherence rate of 94.83%. The two groups were well-balanced at baseline, showing no statistically significant differences (*p* > 0.05) in all characteristics between the two groups (Table 1).

**Table 1:** Baseline characteristics of study participants.

Indicator names	SE egg (*n* = 27)	ZE egg (*n* = 31)	*p-*value
Age (years)	62 ± 9	61 ± 9	0.68
Sex (male/female)	9/18	14/17	0.43
BMI (kg/m^2^)	24.76 ± 2.94	25.14 ± 2.92	0.62
Alcohol consumption, n (%)	8 (29.63%)	7 (22.58%)	0.56
Smoking status, n (%)	8 (29.63%)	3 (9.68%)	0.09
Insomnia, n (%)	13 (48.15%)	10 (32.26%)	0.28
FBG (mmol/L)	13.48 ± 2.26	12.52 ± 2.25	0.11
HbA1c (%)	8.56 ± 1.33	8.88 ± 1.61	0.42
Fasting insulin (mIU/L)	9.89 (7.47, 15.90)	9.40 (5.33, 17.00)	0.42
TG (mmol/L)	1.62 (1.30, 2.74)	2.00 (1.31, 2.64)	0.96
TC (mmol/L)	5.10 ± 0.99	4.81 ± 0.98	0.27
LDL-C (mmol/L)	2.79 ± 0.87	2.48 ± 0.88	0.19
HDL-C (mmol/L)	1.40 ± 0.40	1.42 ± 0.39	0.81

SE egg, selenium-enriched egg; ZE egg, zeaxanthin-enriched egg. BMI, body mass index; FBG, fasting blood glucose; HbA1c, glycosylated hemoglobin A1c; TG, triglyceride; TC, total cholesterol; LDL-C, low-density lipoprotein cholesterol; HDL-C, high-density lipoprotein cholesterol.

### 3.3 Effect of eggs intervention on glycemic metabolism indicators of individuals with diabetes

**Figure 3:**
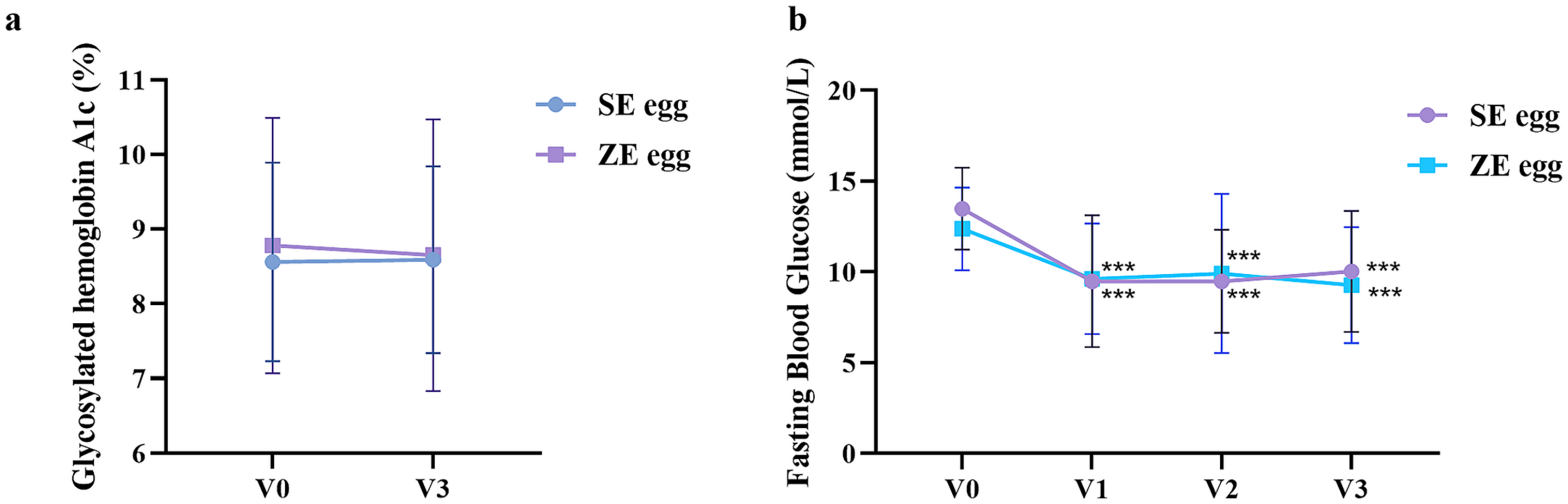
Effects of egg interventions on fasting blood glucose and glycosylated hemoglobin A1c over 12 weeks. **(a)** Glycosylated hemoglobin A1c changes. **(b)** Fasting blood glucose changes.

Both the SE egg group and the ZE egg group exhibited a significant decrease in FBG after 4 weeks which was maintained through to the end of the intervention (Figure 3; *p* = 0.0001). No significant change in HbA1c results was observed in the SE egg group before and after the intervention. Moreover, there was no statistically significant difference in post-intervention HbA1c levels between the two groups.

As shown in Table 2, both groups demonstrated a significant reduction in HOMA-IR (*p* < 0.05). In contrast, the HOMA-*β* index increased specifically in the ZE egg group following the intervention (*p* = 0.06).

**Table 2:** Effects of two types of eggs on glycolipid metabolism in diabetic patients after a 12-week intervention.

Indicator names	Baseline	Post-intervention	*p_0_*	Baseline	Post-intervention	*P_0_*	*P_1’_*	*P_inter_*
Among all individuals								
	SE egg (*n* = 27)			ZE egg (*n* = 31)				
Fasting insulin (mIU/L)	9.89 (7.47, 15.90)	6.73 (4.60, 14.07)	0.04	9.40 (5.33, 17.00)	8.22 (5.64, 17.30)	0.33	0.43	0.27
HOMA-IR	6.16 (4.53, 9.74)	2.79 (1.80, 6.55)	0.006	4.72 (2.63, 9.97)	2.79 (1.80, 6.54)	0.05	1.00	0.46
HOMA-β	20.98 (14.04, 38.01)	27.69 (15.96, 45.77)	0.78	22.48 (13.95, 32.32)	30.50 (16.08, 83.51)	0.06	0.34	0.12
TG (mmol/L)	1.62 (1.30, 2.74)	1.89 (1.14, 2.45)	0.25	2.00 (1.31, 2.64)	1.59 (1.13, 2.47)	0.0007	0.44	0.35
TC (mmol/L)	5.10 ± 0.99	5.31 ± 1.13	0.17	4.81 ± 0.98	4.87 ± 1.11	0.67	0.14	0.43
LDL-C (mmol/L)	2.79 ± 0.87	2.96 ± 0.92	0.22	2.48 ± 0.88	2.63 ± 0.86	0.14	0.17	0.93
HDL-C (mmol/L)	1.40 ± 0.40	1.46 ± 0.43	0.06	1.42 ± 0.39	1.42 ± 0.38	0.92	0.71	0.17
TyG	9.94 ± 0.62	9.54 ± 0.77	0.001	9.80 ± 0.57	9.30 ± 0.72	0.0001	0.22	0.49
Among patients with HbA1c < 8.9%								
	SE egg (*n* = 13)			ZE egg (*n* = 14)				
FBG (mmol/L)	13.19 ± 2.42	7.85 ± 2.08	0.0003	12.11 ± 1.72	7.40 ± 2.62	0.0002	0.63	0.65
HbA1c (%)	7.44 ± 0.69	7.65 ± 0.83	0.14	7.37 ± 0.88	7.29 ± 0.81	0.46	1.00	0.40
Fasting insulin (mIU/L)	14.00 (7.99, 33.25)	7.52 (5.24, 12.33)	0.03	15.00 (8.89, 61.95)	9.48 (6.16, 19.55)	0.08	0.16	0.92
HOMA-IR	7.80 (4.72, 22.5)	2.36 (1.72, 4.90)	0.01	8.74 (4.58, 30.06)	2.67 (1.98, 7.09)	0.02	0.47	0.96
HOMA-β	30.93 (19.94, 59.17)	35.21 (27.15, 51.42)	0.50	31.82 (21.48, 120.92)	71.87 (43.51, 146.77)	0.24	0.06	0.31
TG (mmol/L)	1.61 (1.06, 2.66)	1.93 (1.03, 2.34)	0.49	1.91 (1.39, 2.60)	1.39 (1.13, 2.47)	0.03	0.90	0.47
TC (mmol/L)	5.12 ± 0.70	5.30 ± 0.94	0.49	4.52 ± 0.52	4.46 ± 0.74	0.75	0.18	0.44
LDL-C (mmol/L)	2.80 ± 0.67	2.98 ± 0.77	0.47	2.25 ± 0.45	2.36 ± 0.59	0.39	0.22	0.79
HDL-C (mmol/L)	1.47 ± 0.41	1.51 ± 0.49	0.50	1.39 ± 0.37	1.37 ± 0.34	0.60	0.40	0.39
TyG	9.75 ± 0.50	9.17 ± 0.61	0.006	9.76 ± 0.37	9.03 ± 0.66	0.0005	0.56	0.50
Among patients with HbA1c ≥ 8.9%								
	SE egg (*n* = 14)			ZE egg (*n* = 17)				
FBG (mmol/L)	13.75 ± 2.16	12.06 ± 2.99	0.03	12.87 ± 2.62	11.10 ± 2.76	0.003	1.00	1.00
HbA1c (%)	9.61 ± 0.82	9.45 ± 0.90	0.39	10.13 ± 0.76	9.95 ± 1.31	0.38	0.96	1.00
Fasting insulin (mIU/L)	8.49 (6.37, 14.03)	5.83 (4.06, 18.08)	0.54	5.58 (4.04, 9.71)	6.97 (4.47, 11.87)	0.60	0.94	0.14
HOMA-IR	5.02 (4.09, 8.00)	3.19 (1.80, 10.30)	0.27	3.39 (2.38, 5.10)	2.78 (1.79, 6.42)	1.00	0.66	0.12
HOMA-β	16.90 (13.46, 24.81)	16.35 (10.18, 37.47)	0.71	14.81 (7.03, 23.82)	18.67 (15.65, 30.75)	0.16	0.74	0.16
TG (mmol/L)	1.92 (1.38, 3.26)	1.82 (1.23, 2.85)	0.41	2.27 (0.89, 2.83)	1.75 (0.92, 2.36)	0.02	0.50	0.56
TC (mmol/L)	5.09 ± 1.23	5.32 ± 1.31	0.20	5.06 ± 1.20	5.20 ± 1.27	0.45	0.80	0.73
LDL-C (mmol/L)	2.78 ± 1.04	2.93 ± 1.07	0.27	2.67 ± 1.10	2.86 ± 0.99	0.24	0.84	0.87
HDL-C (mmol/L)	1.33 ± 0.39	1.42 ± 0.38	0.05	1.44 ± 0.42	1.47 ± 0.42	0.51	0.75	0.27
TyG	10.11 ± 0.69	9.88 ± 0.76	0.10	9.84 ± 0.68	9.53 ± 0.70	0.006	0.20	0.66

SE egg, selenium-enriched egg. ZE egg, zeaxanthin-enriched egg. HOMA-IR, Homeostasis model assessment of insulin resistance index; HOMA-β, Homeostasis model assessment of β-cell function index; TG, triglyceride; TC, total cholesterol; LDL-C, low-density lipoprotein cholesterol; HDL-C, high-density lipoprotein cholesterol. TyG, Triglyceride-glucose index; HbA1c, glycosylated hemoglobin A1c. P_0_ indicates the differences before and after intervention within the group. P_1_ indicates the differences between groups after intervention. P_inter_ represents the *p*-value for the interaction effect between group and time.

Table 2 presents the results of subgroup analysis stratified by baseline HbA1c levels (< 8.9% vs. ≥ 8.9%). In the individuals with a baseline HbA1c < 8.9%, both groups exhibited significant reductions in FBG and HOMA-IR. In individuals with baseline HbA1c ≥ 8.9%, observed in both groups were significant reductions in FBG (*p* < 0.05).

Supplementary Table S1 presents the results of subgroup analysis stratified by baseline TG levels (< 1.7 mmol/L vs. ≥ 1.7 mmol/L). Among subjects with normal TG levels at baseline, the FBG (Supplementary Table S1, *p* < 0.01) and the HOMA-IR index (*p* < 0.05) declined significantly in both groups post-intervention. A notable additional finding was that SE egg group demonstrated a greater increase in HOMA-*β* compared to the ZE egg group (*p* = 0.02). Among individuals with abnormal TG levels at baseline, FBG also decreased significantly in both groups after intervention (*p* < 0.01).

### 3.4 Effect of eggs intervention on lipid metabolism indicators of individuals with diabetes

Table 2 illustrates that the ZE egg group experienced a significant decrease in TG (*p* = 0.0007) and the TyG index (*p* = 0.0001). In the SE egg group, only the TyG index showed a significant reduction post-intervention (*p* = 0.001). There were no significant alterations in TC levels in either group post-intervention.

In individuals with a baseline HbA1c < 8.9%, the TyG index declined significantly in both groups after the intervention (*p* < 0.01). Additionally, the TG in group ZE egg declined significantly following the intervention (*p* = 0.03). In the individuals with baseline HbA1c ≥ 8.9%, consistent with the previous subgroup, the TG level (*p* = 0.02) and the TyG (*p* = 0.006) index in group ZE egg still exhibited a significant decrease following the intervention. Notably, the SE egg intervention demonstrated a positive impact on HDL-C levels in this subgroup (*p* = 0.05).

In individuals whose baseline TG levels were within the normal range, a significant reduction in the TyG index was observed in both groups (Supplementary Table S1, *p* < 0.05). In individuals with abnormal TG levels at baseline, TyG index fell markedly in both groups (*p* < 0.01). TG in group ZE egg decreased significantly (Supplementary Table S1, *p* = 0.01). Specifically, HDL-C significantly increased in group SE egg (Supplementary Table S1, *p* = 0.01).

### 3.5 Effect of eggs intervention on diet and exercise of individuals with diabetes

According to the survey data (Supplementary Table S3), there were no significant differences in diet and exercise between the two groups at baseline and post-intervention.

### 3.6 Effect of eggs intervention on safety indicators of individuals with diabetes

After intervention, UA decreased significantly in both groups. Besides, AST in group SE egg increased obviously. Although serum hepatic and renal biomarkers fluctuated slightly within each group after the intervention, all values remained within the normal reference ranges (Supplementary Table S4).

## 4 Discussion

Our study demonstrated that daily consumption of the two types of eggs was effective in improving fasting metabolic profiles and alleviating insulin resistance in patients with T2DM. Moreover, the glucose- and lipid-lowering effects of both egg interventions were more pronounced among participants with a baseline HbA1c < 8.9% or elevated baseline TG levels.

In our study, daily consumption of either egg type significantly decreased FBG and the HOMA-IR index in patients with T2DM. The HOMA-*β* index increased specifically in the ZE egg group following the intervention. Consistent with our findings, Ratliff et al. reported a one-week breakfast regimen including 3 scrambled eggs lowered plasma glucose concentrations in individuals with T2DM (31). In line with this, a separate study reported a significant association between consuming five or more eggs per week and lower fasting blood glucose after 4 years of follow-up (32). We have argued that moving from ~13 mmol/L down to ~10 mmol/L within a short period (V1) and maintaining this level is highly meaningful as a safety signal. It demonstrates that adding two eggs daily does not aggravate glucose toxicity in poorly controlled individuals, and it successfully reduces the total cumulative glycemic load on pancreatic cells. However, this dietary approach cannot replace standard glucose-lowering medications for glycemic normalization. The HOMA-IR index provides a quantitative index of basal insulin resistance in individuals (33). Shirin et al. reported that 12 weeks of daily egg consumption significantly attenuated the HOMA-IR index in T2DM individuals (34). Interestingly, the mechanisms underlying the improvement in HOMA-IR appeared to differ between the two intervention groups. In the SE group, the HOMA-IR reduction was consistent with a significant drop in fasting insulin levels. In contrast, for the ZE group, the improvement in HOMA-IR was largely attributable to the reduction in FBG. This implies that SE egg and ZE egg may act through complementary but distinct metabolic pathways. The HOMA-*β* serves as a validated surrogate marker for estimating pancreatic beta-cell function *in vivo* (35). Another study reported that women who incorporated eggs into their evening meal daily exhibited a significantly higher HOMA-β compared to those consuming egg dishes less than once per week (36). This finding aligns with our results that daily egg intake is potentially associated with enhanced β-cell function. Eggs provide a complete, high-quality source of protein that is digested and absorbed more slowly than carbohydrates, thereby significantly delaying gastric emptying (37), which may partly explain the observed reduction in FBG in diabetic patients following egg consumption. Additionally, eggs contain bioactive components such as selenium and zeaxanthin, both of which possess anti-inflammatory properties and may contribute to improved insulin sensitivity (18, 38). Changes in HbA1c did not reach statistical significance between groups or across the entire cohort after the intervention. This finding is not unexpected given the distinct physiological characteristics of these glycemic markers. HbA1c reflects average glycemic exposure over approximately 8–12 weeks and is therefore less sensitive to short-term metabolic changes, whereas FBG responds more rapidly to interventions that improve hepatic insulin sensitivity (39). In the present study, the intervention duration may have been insufficient to produce substantial or uniform changes in HbA1c, particularly among participants with poorer baseline glycemic control, who often require longer or more intensive interventions to achieve measurable reductions in HbA1c. Additionally, individual variations in red blood cell lifespan or baseline metabolic state within the subgroups might have further diluted the intervention’s impact on this long-term marker. Consequently, while our primary outcome indicates that the intervention did not yield a definitive therapeutic effect on long-term glycemic control, the observed improvements in FBG and insulin resistance indices suggest early, acute beneficial effects on glucose metabolism that may precede detectable changes in HbA1c. These secondary findings should be interpreted with caution due to the negative primary outcome; however, they suggest that moderate egg consumption may be metabolically acceptable and does not adversely affect short-term glucose homeostasis for individuals with impaired glucose metabolism.

In the present study, daily consumption of the test eggs significantly reduced the TyG index in patients with T2DM, and 12 weeks of ZE egg intake additionally reduced TG concentrations. The TyG index is a well-established and clinically applicable surrogate marker for assessing insulin resistance (40). Evidence indicates that a reduction in the TyG index is linked to a lower cardiovascular mortality risk (41). A previous study revealed that when eggs isocalorically replace processed meat in the diet, the TyG index declines by the end of a 156-week follow-up period (42). Nevertheless, the potential association between egg consumption and the TyG index remains underexplored in direct clinical studies. Consequently, our investigation has made some contributions to filling this research gap. Consistent with our findings, Gisella et al. reported a significant reduction in plasma TG concentrations in obese individuals following a 12-week intervention with three eggs per day (43). Similarly, a three-week dietary intervention providing egg-derived protein and unsaturated fatty acids reduced fasting TG levels by 18.5% in overweight or obese adults with baseline hypertriglyceridemia (44). Previous studies have demonstrated that zeaxanthin can significantly reduce serum triglycerides in diabetic rats; moreover, the intake of lutein/zeaxanthin is associated with decreased plasma triglyceride levels in patients with non-alcoholic fatty liver disease. These findings are consistent with the results of the present study, suggesting that the triglyceride-lowering effect observed in the ZE egg group may be partly attributed to its higher concentrations of zeaxanthin (45, 46). Future studies should include plasma carotenoid profiling to establish a formal dose–response relationship. An interesting finding of this study was the significant elevation of HDL-C in the SE egg group. Selenium is essential for the activity of selenoproteins, particularly glutathione peroxidase (47). Elevated selenium intake can enhance the antioxidant capacity of HDL particles, prevent their oxidative degradation and thus maintain higher circulating levels (48). These results suggest that selenium-enriched eggs may offer unique cardiovascular benefits beyond glucose regulation. Notably, the TC levels remained unchanged in diabetic participants after 12 weeks of either egg intervention, indicating that consumption of these test eggs does not impose an additional lipid-metabolic burden in this population.

Furthermore, both egg interventions produced greater improvements in glycemic regulation and lipid profiles among diabetic participants with baseline HbA1c levels below 8.9%. Consistent with our findings, patients with better-controlled baseline FBG achieved a significantly greater reduction in HbA1c after the interventions than those with poorer initial control according to Ayse (49). Similarly, Schwab et al. demonstrated that earlier intervention is associated with more effective glycemic management (50). This enhanced metabolic response is likely attributable to preserved residual *β*-cell function in patients with an earlier-stage of the disease, which in turn sustains a greater capacity to adapt to dietary changes. Among participants with elevated baseline TG, both egg interventions yielded enhanced lipid-lowering efficacy. This augmented hypotriglyceridemic response may be attributed to egg-derived bioactive compounds, such as zeaxanthin, which promote hepatic lipid repartitioning and help restore liver metabolic efficiency (51).

We observed that serum UA levels decreased significantly within the normal range in both groups. This may be related to improved insulin sensitivity (52). Therefore, this finding provides additional supporting evidence for the metabolic benefits of egg intervention. We speculate that components in eggs such as selenium, lutein and zeaxanthin may improve insulin sensitivity and renal excretion function by mitigating oxidative stress and inflammation, ultimately resulting in the observed reduction in uric acid (53, 54). Additionally, the SE egg interventions led to a significant increase in serum AST levels within the normal physiological range in the subjects. This suggests that egg consumption may have activated the systemic metabolic state. Furthermore, given that ALT levels remained stable and all values stayed within the normal range, these changes are unlikely to indicate clinically relevant liver injury.

A notable consideration for this study is the translational feasibility of the intervention. While our results highlight the metabolic benefits of selenium- and zeaxanthin-enriched eggs, the accessibility and affordability of such specialized products for the general population remain practical concerns. However, it is encouraging that agricultural biofortification is rapidly expanding, making functional eggs increasingly available in commercial markets. Furthermore, rather than an immediate general population rollout, this intervention could initially serve as a targeted medical nutrition therapy for individuals with T2DM or pre-diabetes (particularly those with HbA1c < 8.9%). For this specific demographic, incorporating functional eggs into the daily diets may represent a highly cost-effective strategy to prevent further glycemic deterioration. Future translational research and public health initiatives will be essential to make these biofortified foods universally accessible.

This study had several limitations. The predominantly middle-aged and older adult population in this study may limit the generalizability of the findings. Second, no significant differences in HbA1c levels were observed between the groups following the intervention and the subgroup analyses according to HbA1c and TG thresholds were conducted *post hoc* and were not pre-specified. This may indicate that a larger sample size is required to draw more robust and reliable conclusions. Therefore, these results must be interpreted with extreme caution and should be treated strictly as hypothesis-generating rather than confirmatory evidence of differential therapeutic efficacy. While our study only included patients with a diabetes history exceeding 3 years, our data indicates that baseline glycemic severity significantly dictates the intervention’s success. Future studies involving newly diagnosed T2DM patients are warranted to further define the exact early therapeutic window for this dietary approach. Third, our findings are contextualized within the diabetic state, and future studies should include subjects in other metabolic states to further clarify whether the responses differ under different conditions. Fourth, the absence of objective, longitudinal dietary records (e.g., multi-day weighed food diaries or digital photograph-based dietary logs) represents a significant limitation. Consequently, we were unable to rigorously quantify the potential caloric substitution effect-that is, whether the daily intake of the study eggs led to a spontaneous reduction in the consumption of other protein or fat sources. Consequently, this limits our ability to make definitive mechanistic attributions specifically to selenium or zeaxanthin enrichment, as the background nutritional effects of general egg consumption or minor dietary shifts cannot be entirely isolated. Future trials should employ rigorous, continuous longitudinal dietary tracking (such as daily food diaries or controlled metabolic diets) to better isolate the independent effects of bio-fortified micronutrients. Fifth, plasma selenium and zeaxanthin concentrations were not monitored in the current study, which limits our ability to directly correlate the metabolic improvements with systemic micronutrient levels. This remains a hypothesis that requires validation in future studies incorporating pharmacokinetic assessments. Sixth, a fundamental limitation of this study is the absence of a conventional egg control group or a placebo comparator. Because we only compared the two enriched egg formulations against each other, it remains impossible to definitively determine whether the observed improvements in FBG and insulin resistance indices are attributable specifically to the selenium or zeaxanthin enrichment, or merely represent the baseline nutritional effects of general egg consumption itself. Consequently, this direct architectural lack of a true negative control substantially limits our ability to draw strict causal interpretations. Future randomized controlled trials must incorporate a standard, non-enriched egg arm to successfully isolate and validate the independent therapeutic value of these specific micronutrients. Finally, although our sample size was calculated based on the expected HbA1c difference from our preliminary study, the overall change in HbA1c in the full trial did not reach statistical significance. Because the actual observed effect size for overall HbA1c in the full trial was smaller than the anticipated effect size used in our initial calculation, we acknowledge that the study was statistically underpowered to detect a significant difference for this specific primary outcome across the entire cohort. Furthermore, we acknowledge that several secondary outcomes failed to reach statistical significance between the intervention groups. This is primarily because the study’s sample size was computed and powered exclusively for the HbA1c. Therefore, the interpretation of these non-significant secondary outcomes must be approached with extreme caution, and they should be regarded strictly as exploratory findings rather than definitive conclusions. Future large-scale, appropriately powered randomized controlled trials are required to fully elucidate these potential metabolic differences.

## 5 Conclusion

This study demonstrates that while both SE and ZE egg interventions exhibited comparable effects on general glucose metabolism in patients with type 2 diabetes, the ZE egg intervention specifically provided a specific lipid-modulating effect, as indicated by a significant reduction in TG levels. Although overall HbA1c was not significantly improved across the entire cohort, marginal improvement in metabolic control was observed specifically in subjects with a lower baseline HbA1c levels (< 8.9%), suggesting that this dietary intervention is more beneficial when initiated before severe glycemic deterioration.

## Data Availability

The original contributions presented in the study are included in the article/Supplementary material, further inquiries can be directed to the corresponding authors.
