# Supplementary material for: Glycemic and lipid responses to selenium-enriched vs. zeaxanthin-enriched eggs in patients with type 2 diabetes: a 12-week randomized controlled trial
Source: Front Nutr. 2026 Jul 10;13:1803814. doi: 10.3389/fnut.2026.1803814 (PMC13395673; doi:10.3389/fnut.2026.1803814)
Supplement: Supplementary file 2 [file Table_2.docx]

Supplementary Material

Supplementary Note 1: Both groups of hens were fed a base diet of corn and soybean meal. The diet for the SE group was supplemented with insect meal and forage meal, while the ZE group's diet included a specialized mixture of Traditional Chinese Medicine ingredients, such as earthworm, pueraria lobata, and black fungus.

**Table S1** Subgroup analysis by TG status following a 12-week intervention with two egg types on glycolipid metabolism in diabetic patients.

|  | | **SE egg** | | | |  | | **ZE egg** | |  | | |  | |  | |
| --- | --- | --- | --- | --- | --- | --- | --- | --- | --- | --- | --- | --- | --- | --- | --- | --- |
|  | | Baseline | Post-intervention | | *P_0_* | | | Baseline | Post-intervention | | *P_0_* | | *P_1’_* | | *P_2_* | |
| **Among patients with TG < 1.7 mmol/L** | **(n = 14)** | | | | |  | **(n = 13)** | | | | |  | |  | |  |
| FBG (mmol/L) | | 13.68 ± 2.47 | | 9.69 ± 3.95 | | 0.007 | 12.92 ± 2.32 | | 9.50 ± 3.90 | | | 0.001 | | 0.90 | | 0.71 |
| HbA1c (%) | | 8.26 ± 1.40 | | 8.30 ± 1.31 | | 0.77 | 8.66 ± 1.83 | | 8.55 ± 1.97 | | | 0.67 | | 1.00 | | 1.00 |
| Fasting insulin (mIU/L) | | 9.37 (6.95, 27.60) | | 6.08 (3.70, 10.98) | | 0.07 | 9.40 (4.49, 17.15) | | 9.64 (2.69, 17.82) | | | 0.37 | | 0.73 | | 0.29 |
| TG (mmol/L) | | 1.31 (1.06, 1.46) | | 1.16 (1.03, 1.83) | | 0.84 | 1.09 (0.87, 1.45) | | 0.94 (0.73, 1.21) | | | 0.12 | | 0.10 | | 0.41 |
| TC (mmol/L) | | 4.93 ± 0.76 | | 4.95 ± 1.04 | | 0.91 | 4.62 ± 0.52 | | 4.50 ± 0.65 | | | 0.38 | | 0.20 | | 0.56 |
| LDL-C (mmol/L) | | 2.76 ± 0.62 | | 2.75 ± 0.88 | | 0.95 | 2.46 ± 0.49 | | 2.42 ± 0.54 | | | 0.65 | | 0.26 | | 0.87 |
| HDL-C (mmol/L) | | 1.59 ± 0.44 | | 1.60 ± 0.51 | | 0.96 | 1.63 ± 0.46 | | 1.61 ± 0.43 | | | 0.70 | | 0.95 | | 0.73 |
| HOMA-IR | | 5.72 (4.21, 18.33) | | 2.51 (1.40, 4.34) | | 0.04 | 4.74 (2.39, 11.06) | | 2.70 (0.99, 7.56) | | | 0.05 | | 0.81 | | 0.29 |
| HOMA-β | | 22.67 (14.18, 46.67) | | 23.42 (15.14, 41.61) | | 0.58 | 22.97 (10.39, 31.82) | | 20.66 (13.08, 115.65) | | | 0.09 | | 0.85 | | 0.02 |
| TyG | | 9.50 ± 0.31 | | 9.11 ± 0.60 | | 0.05 | 9.34 ± 0.34 | | 8.84 ± 0.57 | | | 0.004 | | 0.24 | | 0.63 |
| **Among patients with TG ≥ 1.7 mmol/L** | **(n = 13)** | | | | |  | **(n = 18)** | | | | |  | |  | |  |
| FBG (mmol/L) | | 13.27 ± 2.08 | | 10.40 ± 2.61 | | 0.001 | 12.24 ± 2.23 | | 9.38 ± 2.80 | | | 0.002 | | 0.31 | | 1.00 |
| HbA1c (%) | | 8.89 ± 1.23 | | 8.89 ± 1.14 | | 1.00 | 9.04 ± 1.47 | | 8.89 ± 1.58 | | | 0.14 | | 1.00 | | 1.00 |
| Fasting insulin (mIU/L) | | 9.98 (7.50, 14.45) | | 7.52 (4.79, 16.39) | | 0.38 | 8.42 (5.58, 15.23) | | 8.09 (6.19, 14.13) | | | 0.71 | | 0.66 | | 0.48 |
| TG (mmol/L) | | 2.74 (2.07, 3.87) | | 2.45 (1.99, 3.55) | | 0.11 | 2.59 (2.25, 2.78) | | 2.15 (1.61, 2.56) | | | 0.01 | | 0.22 | | 0.76 |
| TC (mmol/L) | | 5.29 ± 1.19 | | 5.70 ± 1.12 | | 0.09 | 4.95 ± 1.20 | | 5.13 ± 1.31 | | | 0.36 | | 0.22 | | 0.44 |
| LDL-C (mmol/L) | | 2.82 ± 1.10 | | 3.18 ± 0.95 | | 0.09 | 2.49 ± 1.10 | | 2.79 ± 1.02 | | | 0.06 | | 0.29 | | 0.80 |
| HDL-C (mmol/L) | | 1.18 ± 0.17 | | 1.32 ± 0.26 | | 0.01 | 1.27 ± 0.25 | | 1.29 ± 0.28 | | | 0.61 | | 0.77 | | 0.07 |
| HOMA-IR | | 6.16 (4.63, 7.96) | | 3.59 (2.01, 8.49) | | 0.09 | 4.19 (3.28, 7.56) | | 2.75 (2.30, 6.37) | | | 0.35 | | 0.87 | | 0.52 |
| HOMA-β | | 19.23 (13.91, 36.15) | | 35.21 (15.79, 45.83) | | 0.22 | 22.16 (13.24, 46.72) | | 32.78 (16.06, 83.60) | | | 0.46 | | 0.32 | | 1.00 |
| TyG | | 10.41 ± 0.53 | | 9.99 ± 0.67 | | 0.01 | 10.15 ± 0.42 | | 9.64 ± 0.63 | | | 0.002 | | 0.15 | | 0.64 |

Notes: FBG: fasting blood glucose, HbA1c: glycosylated hemoglobin A1c, TG: triglyceride, TC: total cholesterol, LDL-C: low-density lipoprotein cholesterol, HDL-C: high-density lipoprotein cholesterol, HOMA-IR: Homeostasis model assessment of insulin resistance index, HOMA-β: Homeostasis model assessment of β-cell function index, TyG: Triglyceride-glucose index. *P_0_* indicates the differences before and after intervention within the group. *P_1_* indicates the differences between groups after intervention. *P_2_* indicates the differences in changes between the groups.

**Table** **S2** Changes in metabolic parameters between the SE egg and ZE egg groups.

|  | **SE egg** | **ZE egg** |
| --- | --- | --- |
| **Among all individuals** | **(n = 27)** | **(n = 31)** |
| FBG (mmol/L) | -3.66 ± 3.57 | -3.31 ± 3.13 |
| HbA1c (%) | 0.02 ± 0.62 | -0.14 ± 0.68 |
| Fasting insulin (mIU/L) | -4.28 (-9.52, -0.35) | -2.58 (-4.86, 5.33) |
| HOMA-IR | -2.75 (-6.08, -0.69) | -1.53 (-4.26, 1.15) |
| HOMA-β | -3.05 (-6.15, 15.16) | 1.19 (-4.17, 23.70) |
| TG (mmol/L) | -0.19 (-0.54, 0.21) | -0.20 (-0.59, -0.03) |
| TC (mmol/L) | 0.22 ± 0.79 | 0.06 ± 0.72 |
| LDL-C (mmol/L) | 0.17 ± 0.70 | 0.16 ± 0.57 |
| HDL-C (mmol/L) | 0.07 ± 0.18 | 0.00 ± 0.18 |
| TyG index | -0.40 ± 0.58 | -0.50 ± 0.54 |
| **Among patients with HbA1c < 8.9%** | **(n = 13)** | **(n = 14)** |
| FBG (mmol/L) | -5.34 ± 3.79 | -5.17 ± 3.34 |
| HbA1c (%) | 0.22 ± 0.50 | -0.08 ± 0.40 |
| Fasting insulin (mIU/L) | -5.42 (-10.86, -0.46) | -6.25 (-37.08, -0.90) |
| HOMA-IR | -3.83 (-8.10, -0.81) | -3.89 (-26.33, 0.00) |
| HOMA-β | 0.19 (-5.49, 15.97) | 22.14 (-12.02, 83.65) |
| TG (mmol/L) | -0.21 (-0.54, 0.18) | -0.35 (-0.59, -0.09) |
| TC (mmol/L) | 0.18 ± 0.92 | -0.06 ± 0.63 |
| LDL-C (mmol/L) | 0.18 ± 0.86 | 0.12 ± 0.47 |
| HDL-C (mmol/L) | 0.04 ± 0.19 | -0.03 ± 0.19 |
| TyG | -0.57 ± 0.63 | -0.73 ± 0.60 |
| **Among patients with HbA1c ≥ 8.9%** | **(n = 14)** | **(n = 17)** |
| FBG (mmol/L) | -1.97 ± 2.46 | -1.89 ± 2.11 |
| HbA1c (%) | -0.17 ± 0.68 | -0.19 ± 0.85 |
| Fasting insulin (mIU/L) | -3.54 (-5.38, 2.68) | 0.13 (-2.90, 7.17) |
| HOMA-IR | -2.25 (-4.26, 0.44) | -0.04 (-1.64, 1.15) |
| HOMA-β | -3.48 (-6.15, 13.78) | 1.08 (-3.76, 14.50) |
| TG (mmol/L) | -0.19 (-0.34, 0.21) | -0.21 (-0.55, -0.08) |
| TC (mmol/L) | 0.25 ± 0.66 | 0.15 ± 0.79 |
| LDL-C (mmol/L) | 0.17 ± 0.52 | 0.20 ± 0.65 |
| HDL-C (mmol/L) | 0.10 ± 0.17 | 0.03 ± 0.18 |
| TyG | -0.57 ± 0.63 | -0.74 ± 0.60 |
| **Among patients with TG < 1.7 mmol/L** | **(n = 14)** | **(n = 13)** |
| FBG (mmol/L) | -3.58 ± 4.17 | -3.41 ± 2.98 |
| HbA1c (%) | 0.03 ± 0.54 | -0.09 ± 0.79 |
| Fasting insulin (mIU/L) | -4.28 (-11.26, 2.68) | -2.85 (-6.25, 5.33) |
| HOMA-IR | -2.88 (-8.13, -0.69) | -1.87 (-4.39, 1.15) |
| HOMA-β | -3.86 (-8.52, 15.16) | 15,62 (-3.76, 23.70) |
| TG (mmol/L) | 0.06 (-0.28, 0.27) | -0.18 (-0.35, 0.09) |
| TC (mmol/L) | 0.24 ± 0.83 | -0.03 ± 0.56 |
| LDL-C (mmol/L) | 0.15 ± 0.72 | 0.02 ± 0.43 |
| HDL-C (mmol/L) | 0.03 ± 0.16 | 0.00 ± 0.19 |
| TyG | -0.38 ± 0.68 | -0.49 ± 0.51 |
| **Among patients with TG ≥ 1.7 mmol/L** | **(n = 13)** | **(n = 18)** |
| FBG (mmol/L) | -3.80 ± 1.84 | -3.18 ± 3.46 |
| HbA1c (%) | 0 ± 0.81 | -0.23 ± 0.44 |
| Fasting insulin (mIU/L) | -4.44 (-5.95, -1.05) | -0.26 (-4.44, 3.67) |
| HOMA-IR | -3.26 (-4.32, -1.73) | (-0.93, -3.55, 1.94) |
| HOMA-β | 2.59 (-3.75, 13.73) | 3.51 (-6.37, 29.00) |
| TG (mmol/L) | -0.62 (-1.30, -0.29) | -0.44 (-1.08, -0.14) |
| TC (mmol/L) | 0.16 ± 0.74 | 0.18 ± 0.83 |
| LDL-C (mmol/L) | 0.21 ± 0.68 | 0.36 ± 0.69 |
| HDL-C (mmol/L) | 0.16 ± 0.18 | 0.02 ± 0.18 |
| TyG | -0.41 ± 0.47 | -0.50 ± 0.57 |

Notes: FBG: fasting blood glucose, HbA1c: glycosylated hemoglobin A1c, TG: triglyceride, TC: total cholesterol, LDL-C: low-density lipoprotein cholesterol, HDL-C: high-density lipoprotein cholesterol, HOMA-IR: Homeostasis model assessment of insulin resistance index, HOMA-β: Homeostasis model assessment of β-cell function index, TyG: Triglyceride-glucose index. SE egg: selenium-enriched egg; ZE egg: zeaxanthin-enriched egg.

**Table** **S3** Effects of two types of eggs on exercise and diet in diabetic patients after a 12-week intervention.

|  | **SE egg (n = 27)** | | |  | **ZE egg (n = 31)** | | | |  |  |
| --- | --- | --- | --- | --- | --- | --- | --- | --- | --- | --- |
|  | | Baseline | Post-intervention | | | *P_0_* | Baseline | Post-intervention | *P_0_* | *P_1’_* |
| Steps/day | | 5796.30 ± 2531.57 | 6623.74 ± 4000.15 | | | 0.35 | 5915.48 ± 3071.02 | 6993.55 ±5235.90 | 0.27 | 0.77 |
| physical exercise frequency times/week | | 3.26 ± 1.86 | 3.54 ± 2.00 | | | 0.59 | 3.42 ± 2.23 | 3.31 ± 2.11 | 0.79 | 0.67 |
| **Dietary consumption frequency times/week** | |  |  | | |  |  |  |  |  |
| rice | | 3.30 ± 2.77 | 3.39 ± 2.40 | | | 0.86 | 3.44 ± 2.35 | 2.82 ± 2.25 | 0.10 | 0.36 |
| wheat-based foods | | 5.22 ± 2.15 | 5.04 ± 2.41 | | | 0.72 | 4.40 ± 2.46 | 4.37 ± 2.33 | 0.94 | 0.29 |
| other staple foods | | 1.78 ± 1.60 | 1.75 ± 1.73 | | | 0.70 | 1.90 ± 2.09 | 2.11 ± 2.29 | 0.65 | 0.32 |
| meat | | 3.59 ± 2.61 | 4.17 ± 2.45 | | | 0.33 | 3.94 ±2.56 | 3.74 ± 2.46 | 0.71 | 0.51 |
| poultry | | 1.91 ± 2.05 | 1.70 ± 2.24 | | | 0.53 | 1.19 ± 1.51 | 1.68 ± 2.06 | 0.28 | 0.96 |
| seafood | | 0.30 ± 0.42 | 0.39 ± 0.71 | | | 0.49 | 0.24 ± 0.41 | 0.26 ± 0.51 | 0.88 | 0.42 |
| egg | | 0.57 ± 2.21 | 6.33 ± 1.44 | | | 0.13 | 5.16 ± 2.57 | 5.89 ± 2.31 | 0.11 | 0.38 |
| vegetable | | 6.85 ± 0.53 | 6.70 ± 0.72 | | | 0.16 | 6.45 ± 1.15 | 6.47 ± 1.34 | 0.95 | 0.42 |
| bean products | | 2.93 ± 2.57 | 2.30 ± 1.91 | | | 0.29 | 2.13 ± 2.39 | 1.58 ± 1.97 | 0.30 | 0.17 |
| fruits | | 4.72 ± 2.78 | 5.28 ± 2.37 | | | 0.35 | 4.35 ± 2.77 | 4.24 ± 2.83 | 0.79 | 0.14 |
| dairy products | | 4.15 ± 2.77 | 4.56 ± 2.91 | | | 0.98 | 4.13 ± 2.80 | 3.95 ± 3.00 | 0.12 | 0.82 |

Notes: *P_0_* indicates the differences before and after intervention within the group. *P_1_* indicates the differences between groups after intervention.

**Table S4.** Effects of two types of eggs on liver and kidney function in diabetic patients after a 12-week intervention.

|  | **SE egg (n = 27)** | |  | **ZE egg (n = 31)** | |  |  |  |
| --- | --- | --- | --- | --- | --- | --- | --- | --- |
|  | Baseline | Post-intervention | *p_0_* | Baseline | Post-intervention | *P_0_* | *P_1’_* | *P_2_* |
| AST (U/L) | 20.74 ± 8.24 | 25.59 ± 8.20 | 0.0004 | 22.84 ± 11.39 | 24.10 ± 6.41 | 0.42 | 0.44 | 0.08 |
| ALT (U/L) | 25.85 ± 14.38 | 27.00 ± 13.88 | 0.48 | 26.32 ± 15.52 | 24.78 ± 7.22 | 0.47 | 0.44 | 0.33 |
| UA (μmol/L) | 333.07 ± 89.87 | 294.44 ± 87.83 | 0.005 | 331.13 ± 95.76 | 307.03 ± 83.64 | 0.04 | 0.58 | 0.39 |
| CR (μmol/L) | 66.26 ± 18.90 | 64.44 ± 21.58 | 0.13 | 62.69 ± 14.96 | 61.31 ± 15.98 | 0.08 | 0.54 | 0.75 |
| UR (mmol/L) | 6.20 ± 1.93 | 5.92 ± 1.34 | 0.24 | 6.20 ± 1.33 | 6.26 ± 1.30 | 0.79 | 0.32 | 0.30 |

Notes: AST: aspartate aminotransferase, ALT: alanine aminotransferase, UA: uric acid, CR: creatinine, UR: urea. *P_0_* indicates the differences before and after intervention within the group. *P_1_* indicates the differences between groups after intervention. *P_2_* indicates the differences in changes between the groups. SE egg: selenium-enriched egg; ZE egg: zeaxanthin-enriched egg.

**Table S5.** Baseline distribution of medication uses among groups.

| **Medication** | **SE egg (n = 27)** | **ZE egg (n = 31)** | *P* |
| --- | --- | --- | --- |
| Metformin | 51.85% (14/27) | 61.29% (19/31) | 0.60 |
| Acarbose | 37.04% (10/27) | 16.13% (5/31) | 0.09 |
| DPP4 inhibitors | 7.41% (2/27) | 6.45% (2/31) | 1.00 |
| Insulin | 18.52% (5/27) | 16.13% (5/31) | 1.00 |
| Sulfonylureas | 11.11% (3/27) | 6.45% (2/31) | 0.66 |
| Dapagliflozin | 3.70% (1/27) | 6.45% (2/31) | 1.00 |
| Statins | 18.52% (5/27) | 19.35% (6/31) | 1.00 |
| ARBs | 14.81% (4/27) | 12.90% (4/31) | 1.00 |

Notes: ARBs: Angiotensin II receptor blockers. *P* represents the difference between groups. SE egg: selenium-enriched egg; ZE egg: zeaxanthin-enriched egg.

**Table S6** β coefficients and 95% confidence intervals of FBG and HbA1c trajectories.

| **Population & Model terms** | **FBG** (mmol/L) | | **HbA1c** (%) | |
| --- | --- | --- | --- | --- |
|  | **β coefficient (95% CI)** | ***P*** | **β coefficient (95% CI)** | ***P*** |
| **Among all individuals** |  |  |  |  |
| Intercept | 12.72 (11.29, 14.15) | < 0.001 | 8.82 (8.17, 9.47) | < 0.001 |
| Group (SE vs. ZE) | 0.50 (-1.59, 2.60) | 0.64 | -0.29 (-1.23, 0.66) | 0.55 |
| Time | -0.90 (-1.42, -0.38) | 0.001 | -0.03 (-0.27, 0.20) | 0.78 |
| Group * Time | -0.13 (-0.90, 0.63) | 0.74 | 0.04 (-0.31, 0.39) | 0.82 |
| **Among patients with HbA1c < 8.9%** |  |  |  |  |
| Intercept | 12.10 (9.87, 14.34) | < 0.001 | 7.33 (6.37, 8.29) | < 0.001 |
| Group (SE vs. ZE) | 0.74 (-2.52, 4.01) | 0.65 | 0.08 (-1.32, 1.48) | 0.91 |
| Time | -0.39 (-1.20, 0.42) | 0.34 | 0.62 (0.27, 0.96) | 0.001 |
| Group * Time | -0.26 (-1.45, 0.94) | 0.67 | -0.17 (-0.69, 0.34) | 0.51 |
| **Among patients with HbA1c ≥ 8.9%** |  |  |  |  |
| Intercept | 13.29 (11.51, 15.07) | < 0.001 | 10.12 (9.37, 10.87) | < 0.001 |
| Group (SE vs. ZE) | 0.24, (-2.38, 2.86) | 0.86 | -0.60 (-1.70, 0.50) | 0.28 |
| Time | -1.38 (-2.03, -0.72) | < 0.001 | -0.61 (-0.89, -0.34) | < 0.001 |
| Group * Time | 0.03 (-0.92, 0.99) | 0.94 | 0.25 (-0.16, 0.65) | 0.23 |
| **Among patients with TG < 1.7 mmol/L** |  |  |  |  |
| Intercept | 12.55 (10.06, 15.05) | < 0.001 | 8.63 (7.59, 9.67) | < 0.001 |
| Group (SE vs. ZE) | 0.94 (-2.62, 4.49) | 0.60 | -0.42 (-1.90, 1.05) | 0.57 |
| Time | -0.96 (-1.86, -0.06) | 0.04 | -0.07 (-0.45, 0.30) | 0.71 |
| Group * Time | -0.06 (-1.36, 1.24) | 0.92 | 0.20 (-0.33, 0.74) | 0.46 |
| **Among patients with TG ≥ 1.7 mmol/L** |  |  |  |  |
| Intercept | 12.82 (11.18, 14.47) | < 0.001 | 8.95 (8.13, 9.77) | < 0.001 |
| Group (SE vs. ZE) | 0.11 (-2.36, 2.59) | 0.93 | -0.09 (-1.32, 1.14) | 0.88 |
| Time | -0.83 (-1.44, -0.23) | 0.01 | 0.02 (-0.29, 0.32) | 0.91 |
| Group * Time | -0.19 (-1.09, 0.72) | 0.68 | -0.13 (-0.57, 0.32) | 0.58 |

Notes: FBG: fasting blood glucose, HbA1c: glycosylated hemoglobin A1c, SE: selenium-enriched egg; ZE: zeaxanthin-enriched egg
